# Consistent phenological shifts in the making of a biodiversity hotspot: the Cape flora

**DOI:** 10.1186/1471-2148-11-39

**Published:** 2011-02-08

**Authors:** Ben H Warren, Freek T Bakker, Dirk U Bellstedt, Benny Bytebier, Regine Claßen-Bockhoff, Léanne L Dreyer, Dawn Edwards, Félix Forest, Chloé Galley, Christopher R Hardy, H Peter Linder, A Muthama Muasya, Klaus Mummenhoff, Kenneth C Oberlander, Marcus Quint, James E Richardson, Vincent Savolainen, Brian D Schrire, Timotheüs van der Niet, G Anthony Verboom, Christopher Yesson, Julie A Hawkins

**Affiliations:** 1 School of Biological Sciences, Lyle Tower, University of Reading, Whiteknights, Reading RG6 6BX, UK; 2 Biosystematics Group, Wageningen UR, & Nationaal Herbarium Nederland, Wageningen University branch, Generaal Foulkesweg 37, 6703 BL Wageningen, The Netherlands; 3 Department of Biochemistry, Stellenbosch University, Private Bag X1, 7602 Matieland, South Africa; 4 Institut für Spezielle Botanik, Johannes Gutenberg-Universität Mainz, Bentzelweg 2, 55099 Mainz, Germany; 5 Department of Botany and Zoology, Stellenbosch University, Private Bag X1, 7602 Matieland, South Africa; 6 Royal Horticultural Society Garden Wisley, Woking, Surrey, GU23 6QB, UK; 7 Jodrell Laboratory, Royal Botanic Gardens, Kew, Richmond, Surrey, TW9 3DS, UK; 8 Institute for Systematic Botany, University of Zürich, Zollikerstrasse 107, CH 8008, Zürich, Switzerland; 9 J.C. Parks Herbarium, Department of Biology, Millersville University, Millersville, Pennsylvania 17551, USA; 10 Department of Botany, University of Cape Town, Private Bag X3, 7701 Rondebosch, South Africa; 11 University of Osnabrueck, Department of Biology/Botany, Barbarastrasse 11, 49069 Osnabrück, Germany; 12 The Royal Botanic Garden Edinburgh, 20a Inverleith Row, Edinburgh EH3 5LR, UK; 13 Imperial College London, Silwood Park Campus, Ascot, Berkshire, SL5 7PY, UK; 14 The Herbarium, Royal Botanic Gardens, Kew, Richmond, Surrey, TW9 3AB, UK; 15 UMR C53 PVBMT, CIRAD-Université de la Réunion, 7 chemin de l'IRAT, Ligne Paradis, 97410 Saint Pierre, France; 16 School of Biological and Conservation Sciences, University of KwaZulu-Natal, Pr. Bag X01 Scottsville Pietermaritzburg 3209, South Africa

## Abstract

**Background:**

The best documented survival responses of organisms to past climate change on short (glacial-interglacial) timescales are distributional shifts. Despite ample evidence on such timescales for local adaptations of populations at specific sites, the long-term impacts of such changes on evolutionary significant units in response to past climatic change have been little documented. Here we use phylogenies to reconstruct changes in distribution and flowering ecology of the Cape flora - South Africa's biodiversity hotspot - through a period of past (Neogene and Quaternary) changes in the seasonality of rainfall over a timescale of several million years.

**Results:**

Forty-three distributional and phenological shifts consistent with past climatic change occur across the flora, and a comparable number of clades underwent adaptive changes in their flowering phenology (9 clades; half of the clades investigated) as underwent distributional shifts (12 clades; two thirds of the clades investigated). Of extant Cape angiosperm species, 14-41% have been contributed by lineages that show distributional shifts consistent with past climate change, yet a similar proportion (14-55%) arose from lineages that shifted flowering phenology.

**Conclusions:**

Adaptive changes in ecology at the scale we uncover in the Cape and consistent with past climatic change have not been documented for other floras. Shifts in climate tolerance appear to have been more important in this flora than is currently appreciated, and lineages that underwent such shifts went on to contribute a high proportion of the flora's extant species diversity. That shifts in phenology, on an evolutionary timescale and on such a scale, have not yet been detected for other floras is likely a result of the method used; shifts in flowering phenology cannot be detected in the fossil record.

## Background

Niche conservatism - the tendency of species to retain ancestral limits in tolerances to environmental factors - has a long history [1,2]. From a theoretical perspective, it is supported by the expectation that rates of adaptation of populations to environments outside of the fundamental niche are slow relative to the time to extinction in such environments [3-6]. These assertions based on theory have been followed by a suite of empirical studies that have found evidence both for niche conservatism [7-9] and against it [10-12]. In a recent review, Wiens and Graham [13] conclude that whether niches are conserved or not may simply depend on how similar niches must be to be considered conserved, and that a more productive focus might be on how well the concept of niche conservatism allows us to predict outcomes in different areas of ecology and evolution.

Here we use a phylogenetic approach to investigate changes in distribution and flowering phenology of a species-rich flora through a period in which it was exposed to numerous climatic changes of a consistent and repetitive nature, and compare how organisms that responded in different ways have contributed to extant species diversity. Our study uses phylogenies representing 20% of the extant angiosperm flora of the Cape of South Africa (1800 of the 8900 species), a biodiversity hotspot that has experienced climatic changes associated with strong seasonal aridification since the Neogene (*ca. *2-14 million years ago), likely in several different episodes [14].

Elsewhere in the world, at an intra-specific level, a plethora of past studies have found evidence for local adaptation to contemporary climate change at specific sites [15], although some dispute this evidence in all but a minority of cases [16,17]. However at the level of species or evolutionarily significant units, the traditional view is based on the fossil record [18-20], which shows that in response to past climatic changes in the Quaternary (fluctuations occurring on the timescale of thousands of years, within the last 2.6 million years) species underwent dramatic distributional shifts, but retained remarkable stability in phenotype and inferred ecology. The importance of distributional shifts on this timescale is further supported by the success of ecological niche models in predicting ancient distributions [21,22].

Over timescales of millions of years, the documented response of floras to past environmental change is that of species-level turnover [23,24]. It is likely that there is a strong adaptive component to such turnover, but an understanding of the relative roles of adaptation and species sorting is complicated by the fact that the timescale of change (hundreds of thousands to millions of years) corresponds to the typical duration of species; many of the characters detected in the fossil record that may exhibit adaptive responses to past climatic change (e.g. leaf micromorphological features such as stomatal density and distribution, and trichome abundance) are themselves used for species delimitation. While flowering phenology is subject to strong physiological constraints and therefore likely to be strongly linked to climate, it cannot be inferred from the fossil record, regardless of timescale. Reconstructions based on molecular phylogenetic data offer certain advantages over the fossil record with respect to these issues. Notably, the characters used for lineage delimitation are different from those used to detect adaptive responses to climate change. Further, it is possible to include in an analysis both a wide breadth of lineages, including those absent from the fossil record, and any character identifiable across their living representatives.

**Figure 1:**
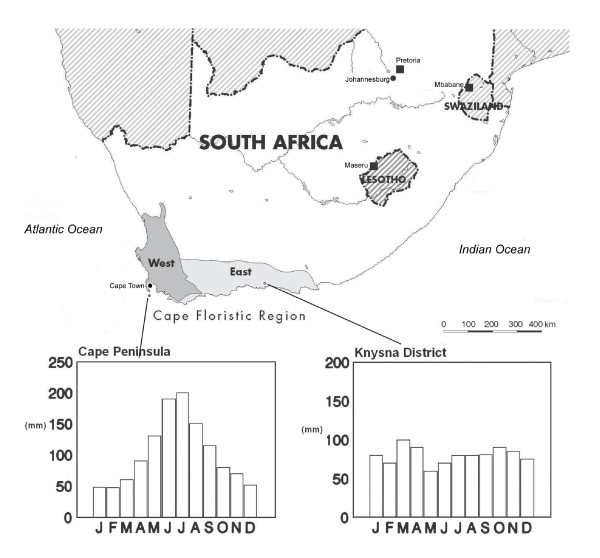
**Location of the Cape Floristic Region in southern Africa as defined by Goldblatt & Manning **[25]. Western and eastern regions with winter and nonseasonal rainfall regimes, respectively, are indicated, along with representative seasonal rainfall charts (after [84]).

The species-rich Cape Floristic Region (CFR) provides a model system in which to assess the utility of niche conservatism in explaining floral responses to past climatic change. Firstly, the concentration of the Cape's floral species diversity within a small number of unusually large endemic or near-endemic radiations permits the development of robust phylogenetic hypotheses through intense sampling focused in a small geographic area. Consequently, molecular phylogenetic coverage of plant lineages in this biota is more complete than that of any other biodiversity hotspot. Secondly, despite being unusually species-rich both for its area and latitude, the entire seed-plant flora has been catalogued, with detailed locality and phenological information available for each species [25]. Thirdly, the CFR spans two regions with a major difference in climatic regime (Figure 1). The eastern region has a nonseasonal rainfall climate, while the western region has a strictly winter rainfall (Mediterranean-type) climate. Evidence from global climate data support a nonseasonal rainfall climate across the Cape in the Early-Mid Miocene [26,27]. This was followed by a seasonal aridification trend along the west coast [14], the precise timing of which is uncertain, in which summer rainfall was reduced and winter rainfall was increased. Evidence from fossil data supports numerous later fluctuations between non-seasonal rainfall and winter rainfall regimes in the western region, culminating in the modern winter rainfall regime in the western region [28-34]. However the number and precise timing of these fluctuations remain uncertain.

The current flora of the winter rainfall region shows a peak of flowering in the spring (September-October), while that of the nonseasonal rainfall region shows a peak in late-spring/early summer (October-November)[35]. Species in the winter rainfall region also show a shorter mean duration of flowering (3.21 months, n = 3670 for all seed plant species; 3.05 months, n = 877 for all species in clades sampled) than species confined to the east (4.04 months, n = 2316 for all seed plant species; 3.56 months, n = 373 for all species in clades sampled; [25]). These two patterns are consistent with expectations if insufficient moisture for physiological activity is the main factor limiting summer flowering in the CFR [35]. Species occupying the Cape-wide nonseasonal rainfall regime prior to the summer aridification trend, and that were physiologically vulnerable to the new climate, could have survived by adapting to the new climate, by changing their distribution, or a combination of the two. Based on the broad pattern of aridification since the Mid Miocene, the predicted direction of adaptive changes in phenology would be from long-duration summer flowering to shorter-duration spring flowering. This predicted directionality holds regardless of whether adaptive changes are the result of fixed genetic changes or plastic responses. In terms of distribution, reduced survival in the west, and distributional shifts towards the east where the effects of summer aridification were less severe, would also be predicted.

We compiled molecular phylogenies representing 20% of the angiosperm species diversity of the CFR [36-50] as defined by Goldblatt and Manning [25]. These 18 molecular phylogenies represent monophyletic floral clades largely or entirely restricted to the Cape, estimated to have diversified within the last 46 Myr [39,40,42,45,47,48,50-55]. Published data [25] for geographical distributions and for the timing and duration of flowering of extant species were used to reconstruct maximum likelihood (ML) estimates of the ancestral states of these clades.

Our aim was to test the relative contribution of distributional shifts and adaptive change in the response of the Cape flora to climatic changes since the mid-Miocene. If niche conservatism predominates, then lineages must track the non-seasonal rainfall regime from the West to the East in order to continue to occupy a physiologically favourable environment. We therefore expect lineages whose ancestors experienced climate change in the West to show distributional shifts to the East.

A species' niche is the multidimensional set of biotic and abiotic conditions in which it is able to persist and maintain stable population sizes. For a flowering plant, one aspect of its overall niche is its flowering phenology - i.e. the timing and duration of its flowering period. Since flowering is known to be limited physiologically by many aspects of climate [56], we consider flowering phenology to be one of the suitable dimensions of niche space in which to look for adaptive shifts in response to past changes in climate in the Cape. Adaptive changes in phenology could considerably modify species climate envelopes, but not necessarily accompany a shift of biome or vegetation type. Whether adaptive changes result from fixed genetic changes or plastic responses is best determined by experimental transplantation of plants, which has not yet been carried out in the Cape. These two mechanisms cannot be distinguished on the basis of phylogeny alone. Nonetheless, the identification of the scale of such changes using phylogeny can serve as a stimulus for future experimental work. In screening the Cape flora for changes consistent with predictions under past seasonal aridification, we find a number of distributional shifts. Surprisingly however, shifts in flowering phenology of lineages consistent with predictions also occur, are similar in frequency to distributional shifts, and belong to lineages that have proceeded to contribute a large proportion of species-diversity to the extant flora.

## Results

**Figure 2:**
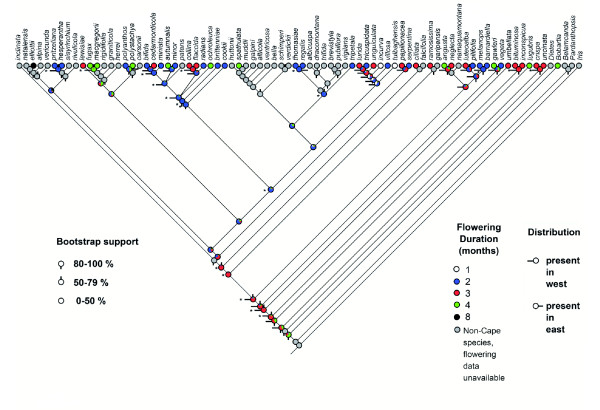
**Phylogeny of *Moraea *showing reconstructed shifts in flowering duration and distribution under an ML model**. All species are in the genus *Moraea *except where otherwise indicated. Coloured balls at terminals indicate the flowering duration of species, while equivalent coloured pie diagrams at each internal node depict the proportional likelihood for different flowering durations. One month, white; 2 months, blue; 3 months, red; 4 months, green; 8 months, black. Nodes reaching the threshold of two log-likelihood units separating the flowering duration of highest likelihood from alternative flowering durations are marked with an asterisk. Bars attached to the left side of nodes indicate significant support (two log-likelihood units separation) for a reconstructed presence in the west; those to the right side of nodes indicate significant support for a reconstructed presence in the east. Internal nodes with no horizontal bars are those for which the reconstructed distribution is not significantly supported. A vertical bar below a node indicates 80-100% bootstrap support; a bar above the node indicates 50-79% bootstrap support; no vertical bar indicates 0-49% bootstrap support.

Our phylogenetic reconstructions demonstrate that 9 clades have undergone shifts in flowering pattern consistent with predictions based on past climate change (Figure 2; Table 1). How the number of shifts per Cape clade are counted depends on the treatment of nodes for which character states are unresolved; we use the basal-most possible position of shifts (as in additional file 1: AF1.pdf) in order to produce figures that are comparable across Cape clades. On this basis, shifts in flowering pattern consistent with predictions consist of one (Figure 2, and see AF1.pdf, Trees 5, 8, 10, 12 &13), two (AF1.pdf, Tree 6) or four (AF1.pdf, Tree 5) shifts in flowering duration per Cape clade, and one (AF1.pdf, Trees 3, 5, 6 & 9) or two (AF1.pdf, Tree 5) shifts in the timing of flowering per Cape clade, making a total of eleven shifts in flowering duration and six shifts in the timing of flowering detected across the sampled flora. By contrast, four clades have undergone shifts in flowering pattern contrary to predictions (Table 1, additional file 2: AF2.pdf); these consist of one (AF1.pdf, Trees 5 & 10) and two (AF1.pdf, Tree 14) shifts in flowering duration per Cape clade, and one shift in the timing of flowering per Cape clade (AF1.pdf, Trees 5, 10 & 12), making a total of four shifts in flowering duration and three shifts in the timing of flowering contrary to predictions detected across the flora.

**Table 1:** Phenological and distributional patterns in the eighteen Cape clades sampled.

Cape Clade	Shift in flowering midpoint	Shift in flowering duration	Shift in distribution	Date estimate (Ma) and method(s) used
Bruniaceae			✓	6.7-4 clock [55]

Crotalarieae	✓^1^	✓ ^b^	✓^b^	40-8.8 NPRS, multidivtime [39,52]

*Disa*	✓		✓	27-1 multidivtime [53]

*Ehrharta*				

*Ficinia*			✓	

*Heliophila*	✓	✓		5.8-1.0 NPRS, clock [45]

*Indigofera*			✓	11-5.5 PL [85]

*Moraea*		✓	✓	14-4 NPRS [42]

*Muraltia*	✓		✓	12.7-0.9 multidivtime [40]

*Oxalis*	^3^	✓^b^	✓ ^b^	

*Pelargonium*			^2^	

*Pentaschistis*	^5^	✓	✓	

*Phylica*		✓	✓	8-2 NPRS [47]

Podalyrieae	✓	✓		40-10 NPRS, multidivtime [39,52]

Cape Restionaceae		^4^	✓	42-1.25 NPRS, clock [54]

*Satyrium*				

*Tetraria*			✓^b^	

*Zygophyllum*				

For each Cape clade in question, ticks (✓) indicate one or more supported shifts in character state consistent with our past climatic change predictions. Unless otherwise indicated, shifts in flowering midpoint are from the summer towards the spring, shifts in flowering duration are reductions in the number of months of flowering, and shifts in distribution are out of the west and/or into the east. Where available, ranges of published date estimates for the shifts are listed, along with the dating methods included in this range and reference to source publications.

✓^b^, clades in which some degree of later backward shifting is exhibited at the distal-most nodes. ✓^1^, in the Crotalarieae, the flowering midpoint is reconstructed as shifting from summer to early spring deep within the Cape clade, followed by a much shallower shift to late spring and finally back to early spring. A reduction in flowering duration is reconstructed both deep within the Cape clade, and towards the tips. 2, *Pelargonium *exhibits a shift from east to west at a single distal-most node. 3, *Oxalis *exhibits a shift in reconstructed flowering midpoint from mid-May to the May-June boundary and/or to mid-June (i.e. from late-autumn slightly towards the winter). 4, the Cape Restionaceae exhibits a shift in reconstructed flowering duration from one to two months, and from one to five months at a single distal-most node. In the only instance where such shifts do not involve very shallow nodes, a later shift back to one flowering month is observed. 5, Depending on the nodes considered either side of those for which states are undetermined, *Pentaschistis *either exhibits a very slight shift in flowering midpoint from summer towards the spring, or an equally slight shift from spring towards the summer.

In terms of distribution, 12 clades show predicted shifts from the west towards the east (intra-Cape shifts out of the west, into the east, or a combination of the two; Figure 2, Table 1); these consisted of one (AF1.pdf, Trees 2, 10 & 13), two (Figure 2; AF1.pdf, Trees 1, 3, 7, 8, 9 & 12), three (AF1.pdf, Tree 5) and seven (AF1.pdf, Tree 14) shifts out of the west and/or into the east per Cape clade, making a total of 26 shifts in distribution detected across the sampled flora. By contrast, four clades show distributional shifts from the east towards the west, contrary to predictions (intra-Cape shifts out of the east, into the west, or a combination of the two; Table 1, AF2.pdf); in all cases these consisted of just one (AF1.pdf, Trees 2, 5, 10 & 11) shift out of the west or into the east per Cape clade, making a total of four shifts in distribution contrary to predictions detected across the flora.

Thus, of the 18 clades sampled in our study, almost as many clades show shifts in flowering phenology consistent with adaptation to climate change (9 clades) as show changes in distribution (12 clades). Seven clades showed shifts in both phenology and distribution consistent with predictions. A smaller number of clades show shifts in flowering phenology and distribution contrary to adaptation to climate change (4 clades in each case, of which two show both reverse patterns).

We also counted the number of Cape species in clades that speciated following phenological and distributional shifts, in order to determine the scale of contribution of such lineages to the extant flora. When species numbers in our sample are considered, and errors caused by unsampled species and uncertainty in the exact node of character state transition are allowed for, 14-55% of species belong to lineages that have experienced a shift in phenology consistent with an adaptation to climate change. By comparison, our reconstructions indicate that 14-41% of species have arisen from ancestors that showed a distributional shift consistent with climate change.

## Discussion

The reproductive output of plants depends on the fine-tuning of flowering to fit abiotic and biotic conditions [57,58]. Thus flowering phenology has strong fitness consequences, and flowering time is one factor determining a species' niche [59]. Like other characters specifying a species' niche, physiological or morphological, flowering phenology may be conserved, plastic or undergo evolutionary change [57,59].

It is well established that many plant populations can and have changed their flowering time in the last century. Shifts in flowering phenology provide some of the most compelling evidence that species are being influenced by contemporary global environmental change [60-62]. These tracking responses, perceived as adaptations to changing environmental conditions, may be environmentally induced plastic responses or evolutionary adaptations. Although genetic data is not available for most of the species showing phenological responses to climate change [17], there are several studies which have demonstrated that responses can be heritable. These include examples of crop plants responding to environmental changes *in situ *and invasive plants or domesticates encountering new climate regimes as they expand their distribution [63-67]. Differential tracking of contemporary climate change has been shown to be a determinant of the species composition of a community, since populations of species lacking a plastic or microevolutionary response are locally extirpated [68,69]. These findings raise questions about the significance of phenological adaptation to past climate change, since the extent to which differential tracking of past climate has shaped a whole flora is at present unknown.

Our study has identified a footprint of past phenological change, set in motion over five million years ago, in a contemporary flora. A point that remains clear regardless of the relative roles of evolutionary changes and phenotypic plasticity is that the observed phenological shifts have had a major role in shaping the extant Cape angiosperm flora; lineages that underwent shifts in flowering phenology later speciated extensively. Our study also provides tentative evidence of differential tracking of climate change, with phenological shifts apparent in some clades but not others.

The comparable number of shifts - distributional and phenological - experienced by the Cape flora as a new climate regime was established is a noteworthy finding of our study. Table 1 shows that phenological shifts are apparent in some clades (Crotalarieae, *Disa*, *Heliophila*, *Moraea*, *Muraltia*, *Oxalis*, *Pentaschistis*, *Phylica *and Podalyrieae) but not others. We go beyond estimation of the frequency of shifts to estimate the scale of the contribution these shifts have made to the character of the present-day flora. While our confidence intervals are wide - 14-41% and 14-55% of species belonging to lineages that have experienced distributional and phenological shifts consistent with past climate change, respectively - these figures indicate that contrary to certain schools of thought [18,20], adaptive changes consistent with past climate change have had a significant impact on the Cape flora on the timescale considered (Early-Mid Miocene to the present). Such adaptations may be close contenders to (often co-occurring) distributional shifts in their frequency and contribution to the modern flora. Our results, while suggestive of evolutionary changes, do not allow us to rule out a major role for plasticity in the phenotypic adaptations that we observe. Furthermore, flowering phenology is just one of many important niche parameters of Cape plants, most of which are presumably highly conserved, otherwise there would not be large clades restricted to the Cape (Cape clades), and the Cape Floristic Region as we know it would not exist. Nonetheless, the patterns uncovered demonstrate that both adaptive and distributional shifts consistent with past climatic change have had strong impacts on the assembly of this biodiversity hotspot, since lineages that underwent these changes went on to contribute a high proportion of its current species richness.

While most of the observed shifts in distribution and flowering patterns are consistent with predictions based on past climate change, alternative explanations must be considered. First, we consider the possibility that reconstructed shifts in flowering patterns result from changes in distribution within the Cape, with a consequent phenological response to regions with differing climates, rather than climatic change itself. This scenario can be rejected because reconstructed shifts in flowering pattern (long-duration summer flowering to shorter-duration spring flowering) work in the opposite direction to predictions on the basis of reconstructed west to east shifts in geography.

Second, we consider the possibility that reconstructed shifts are an artefact of regional differences in species diversity within the Cape; the west is known to harbour higher species diversity than the east [14]. Were the flowering duration or season of Cape species distributed at random with respect to phylogeny, we might expect the predominant flowering states observed in the west (short-duration spring flowering) to be more frequently reconstructed at the base of Cape clades than those observed in the east (longer-duration summer flowering), purely as a result of them being the most frequent in the dataset. This second scenario can be rejected, since observed shifts in flowering phenology run in the opposite direction to those predicted as a result of the proposed diversity difference artefact.

Third, we consider the possibility that the patterns we observe have arisen by chance. Our character randomizations of flowering midpoint across trees showing the basal-most (Podalyrieae) and distal-most (*Disa*) shifts in flowering midpoint showed that the probability of patterns consistent with past climatic change occurring by chance are P = 0.00 and P = 0.1 respectively. If we take the higher of these two probabilities as representative of the maximal probability of shifts consistent with past climatic change occurring by chance in any particular Cape clade, and consider the five out of 18 Cape clades for which shifts consistent with past climatic change arose in the real data, we can reject the hypothesis that the observed shifts occurred by chance (P = 0.0218).

It is appealing to link environmental changes and shifts in phylogenies through estimations of the timescales on which they occurred. In the case of the seasonal aridification trend in the Cape flora, problems with such an approach arise. The most significant of these is the absence of data concerning the precise timing, frequency and nature of the aridification trends; much more palaeobotanical and geological evidence is needed if we are to narrow down their timing reliably within the broad bounds of the Neogene. Notwithstanding this caveat, in all sampled Cape floral clades for which date estimates are available, confidence intervals for the timing of distributional and phenological shifts strongly overlap with temporal bounds of the aridification event (Table 1).

Since our phylogenies only sample living species, we are unable to speculate on any responses of non-surviving lineages of the early flora prior to their extinction. Clearly however, our conclusions regarding the type of response of lineages that survived past climatic change are unaffected by the type of response (or lack of response) of lineages that became extinct. Further to complete extinction, two patterns of local (regional) extinction may be envisaged. The first is extinction within the CFR with the lineage surviving as a "non-Cape relative" outside the CFR; we refer to this as 'Cape departure'. The second is relatively rapid back-and-forth shifting of distribution, leaving no significant distributional change between start and finish; we refer to this as 'back-and-forth shifting'.

Cape departure seems unlikely to present a major distortion of the broad-scale pattern presented here; such cases are infrequent judging by the small number of complete departures from the CFR occurring within the monophyletic (or nearly so) groups of Cape species ('Cape floral clades'). Therefore compared with most continental floras, the CFR can be viewed as being close to a closed system. Under the back-and-forth shifting scenario, a lineage's temporary absence accompanied by a shift in phenology and reinvasion of the CFR could have been misinterpreted as *in situ *phenological change. However, we have to invoke local extinction in the Cape at the time that the lineage colonized the neighbouring region, followed by local extinction in that neighbouring region as the lineage re-colonized the Cape. While this is not impossible it should first be noted that virtually any interpretation of a phylogeny can be refuted if enough extinction events in the right place are hypothesised. The more extinction events that are needed, and the more specific the placement of the extinct species required, the less strong such counter-arguments may seem. Here we require a minimum of two for each Cape clade inferred to undergo a phenological shift, making 18 local extinction events in total. More importantly, this scenario still involves phenological shifts. The only difference is that the phenological shifts occur while the lineages are outside the Cape, rather than being *in situ*. This would be an important detail to note were evidence found in favour. However, our main conclusion - that a large proportion of Cape clades have undergone phenological shifts during a period of past climatic change - would remain unchanged.

The cases of phenological shifts that we document provide a highly conservative estimate of the likely frequency of niche shifts in the Cape flora overall; we investigated shifts in timing and duration of flowering only, and there are many other pathways by which plants may have survived summer aridification events. These include the evolution of an annual life form, and changes in leaf longevity, sclerophylly, leaf size, root depth and root storage, all of which are interpreted by plant physiologists as mechanisms of drought resistance [70-74]. Improvements in a species' capability to survive a higher incidence of fire [14] through resprouting or reseeding may also have been important. While many of these species traits might be ideal for testing for adaptation to aridification between closely related species, few if any of them are likely to have been as important responses across the taxonomic breadth of the Cape flora as is flowering phenology.

## Conclusions

Adaptive changes in ecology at this scale and consistent with past climate change have not been documented for other floras. For example, palynological studies, most complete for the Northern hemisphere, suggest that many plant species underwent major distributional shifts in response to Late Quaternary climatic fluctuations but appear to have remained unchanged in phenotype [18,20]. The same applies to beetles [19]. Clearly however, such studies compare the external morphology of living and fossil forms; on this basis, nothing can be inferred about the stability or otherwise of characteristics such as phenology over this timescale.

Despite concordance with conclusions based on the Quaternary fossil record (short timescales of 10^3 ^to 10^4 ^years), the phenomenon of niche conservatism initially came as a surprise and paradigm shift to those familiar with strong patterns of adaptive change in response to biotic and abiotic changes over long timescales in the fossil record (10^4^-10^9 ^years, e.g. [75]). Nonetheless, this paradigm and associated distributional shifts gain support from several lines of inference, including the success of ecological niche models in predicting geographical distributions, most notably in reciprocal comparisons between the Last Glacial Maximum (based on fossils) and the present [21,22]. Such studies have provided a picture of species' geographic distributions tracking particular climatic regimes closely [76]. Ecological niche models involving climate have also shown strength in predicting the geography of species invasions in different geographical settings [77], and in predicting distributions of sister species separated by several million years of independent evolution [8]. Finally, a wealth of population genetic studies have supported the conclusion that species underwent distributional shifts in response to past climate change [78,79].

In terms of adaptive responses, numerous ecological studies provide evidence for intraspecific adaptation to current and past (Late Quaternary) climate change at specific sites, predominantly in the northern hemisphere [64,78-80]. The absence of occurrence of novel phenotypes across a whole species or evolutionarily significant unit through periods of past climate change [15], as inferred from the Quaternary fossil record, has recently been questioned from palaeoecological perspectives [81]. However it has remained unchallenged by phylogenetic studies. Given the high frequency of Cape lineages that underwent phenological responses consistent with past seasonal aridification, and the difficulty of recovering such shifts from the fossil record, it is conceivable that such shifts in niche through periods of past climate change have been more prevalent than is currently appreciated. Further, the importance of shifts in ecology [15], in addition to range shifts [18], in the Cape flora's response to past climatic changes may have been underestimated. In addition to the limitations of the fossil record in preserving ecological shifts, such shifts may be more easily detected at the species level over the course of longer timescales (10^4^-10^9 ^years, for which fossil data is not available in all biomes) than shorter ones (10^3 ^years). Considering the ongoing accumulation of molecular phylogenetic data worldwide, we hope that our findings will stimulate comparable investigations across other biomes for which the timing and nature of past climatic changes have been more precisely documented.

## Methods

### Species sampling

Phylogenies of 18 floral clades largely or exclusively restricted to the Cape were compiled from existing publications [36-50]. These clades vary in the number of Cape species they include, from 20 to an estimated 378 species, cumulatively totalling 20% of the flora's species diversity. Cape clades were identified following Linder's [14] criteria. Sampling of Cape species within these clades varied from 100% to 10% per phylogeny. Tree topologies used for analysis were either one of the most parsimonious trees, or the Bayesian tree gaining highest posterior probability, with the exception of five Cape clades for which only a consensus tree was available from the source publication.

### Character scoring

Flowering duration and the midpoint of flowering season were treated as separate characters and scored for each species based on Goldblatt & Manning's [25] data on flowering period in months. Flowering duration was scored with 12 states corresponding to number of months, and midpoint of flowering was scored with 24 states corresponding to mid-points that fall either mid-month or at the transition between two months. Three species had a split flowering season unlikely to result from a paucity of records, and their flowering durations and midpoints were consequently scored as missing data. Distributional data were available in the form of presence or absence in each of six phytogeographic centres that Goldblatt & Manning [25] used to subdivide the Cape. Given the proximity between the boundaries of these centres and the division of Johnson's [35] two rainfall regions, we scored species as present or absent from Goldblatt & Manning's [25] western (NW, SW) and eastern (KM, LB, AP, SE) phytogeographic centres, corresponding to Johnson's [35] winter rainfall and nonseasonal rainfall regimes respectively.

### Reconstruction of ancestral states and state shifts

Ancestral states were reconstructed using a single-rate maximum likelihood (ML) model implemented in Mesquite version 1.11 [82]. The advantage of this approach is that it permits an estimate of the uncertainty in state reconstruction [83]. A minimum of two log-likelihood units separating a single state of highest likelihood from alternative states was used as a threshold for determining the state optimisation of each internal node. Optimisations at nodes for which this threshold is not reached, or for which two log-likelihood units separate more than one state of high likelihood from others, were considered undetermined. Where nodes optimised as different states are separated by nodes in which the ancestral state is undetermined, we quote species diversities under the two extremes (deepest and shallowest) of potential location of the state shift. Likewise, we quote species diversities of clades under the two extremes of placement of unsampled species. To calculate the probability of shifts consistent with past climatic change occurring by chance, we carried out randomizations in which we permuted the observed taxa among the terminal nodes in any given tree using the Reshuffle Terminal Taxa option in the TreeFarm package of Mesquite. Fifty randomizations of flowering midpoints were conducted across two Cape clades selected to represent trees with the basal-most and distal-most placement of flowering midpoint shifts. The resulting 100 trees were manually examined for shifts consistent with past climatic change, following the same criteria that were used in the observed data.

## Authors' contributions

BHW and JAH conceived and designed the study; FTB, DUB, BB, RC, LLD, DE, FF, CG, CRH, HPL, AMM, KM, KCO, MQ, JER, VS, BDS, TV and GAV provided phylogenetic data and feedback on results; CY reformatted phenological and distributional data; BHW coordinated data assembly and conducted all analyses; BHW and JAH wrote the manuscript; all authors edited and approved the final manuscript.

## Supplementary Material

Additional file 1 **Molecular phylogenetic trees with reconstructed shifts in geographic distribution and flowering patterns (flowering durations and flowering midpoint) indicated**. Unless otherwise indicated, shifts in flowering patterns are in the direction consistent with past climatic change; shifts in flowering midpoint are from the summer towards the spring, and shifts in flowering duration are reductions in the number of months of flowering. Where nodes optimised at different states are separated by nodes in which the ancestral state is undetermined, we have marked on the basal-most possible location of the shift. Click here for file

Additional file 2 **Table illustrating the degree of shift in flowering phenology in the eighteen Cape clades sampled**. Mid-month flowering midpoint character states are indicated by an abbreviation for the month in question, while character states at the boundary between two months are indicated by those two month abbreviations separated by a hyphen. Flowering durations are in months. Shifts from the base of the tree towards the tips are indicated as ">". Note that all possible series in the degrees of shift of flowering midpoint and duration are listed. Many nodes optimised at different states are separated by nodes for which the ancestral state is undetermined. Therefore, how many shifts are counted depends on the criteria used to count them. In order to be conservative in our counting, we have counted shifts in the text, in Table 1 and in additional file AF1.pdf based on the basal-most possible location of each shift. As a result of these criteria and tree shape, there are many more possible series in the degrees of shifts marked here than there are basal-most possible positions of shifts in the text, Table 1 and file AF1.pdf. Click here for file
