# Supplementary material for: Consistent phenological shifts in the making of a biodiversity hotspot: the Cape flora
Source: BMC Evol Biol. 2011 Feb 8;11:39. doi: 10.1186/1471-2148-11-39 (PMC3045326; doi:10.1186/1471-2148-11-39)
Supplement: Additional file 1 — Molecular phylogenetic trees with reconstructed shifts in geographic distribution and flowering patterns (flowering durations and flowering midpoint) indicated. Unless otherwise indicated, shifts in flowering patterns are in the direction consistent with past climatic change; shifts in flowering midpoint are from the summer towards the spring, and shifts in flowering duration are reductions in the number of months of flowering. Where nodes optimised at different states are separated by nodes in which the ancestral state is undetermined, we have marked on the basal-most possible location of the shift. [file 1471-2148-11-39-S1.PDF]

Additional file 1 – Molecular phylogenetic trees with reconstructed shifts in geographic distribution and flowering patterns (flowering durations and flowering midpoint) indicated.

Unless otherwise indicated, shifts in flowering patterns are in the direction consistent with past climatic change; shifts in flowering midpoint are from the summer towards the spring, and shifts in flowering duration are reductions in the number of months of flowering. Where nodes optimised at different states are separated by nodes in which the ancestral state is undetermined, we have marked on the basal-most possible location of the shift.

Bruniaceae (1 Cape clade): Tree 1

Cyperaceae (*Ficinia* & *Tetraria*): Tree 2

Disa (1 Cape clade): Tree 3

Ehrharta (1 Cape clade): Tree 4

Genistoids (Crotalarieae & Podalyrieae): Tree 5

Heliophila: Tree 6

Indigofera: Tree 7

Moraea: Tree 8

Muraltia: Tree 9

Oxalis: Tree 10

Pelargonium: Tree 11

Pentaschistis: Tree 12

Phylica: Tree 13

Restionaceae: Tree 14

Satyrium: Tree 15

Zygophyllum: Tree 16

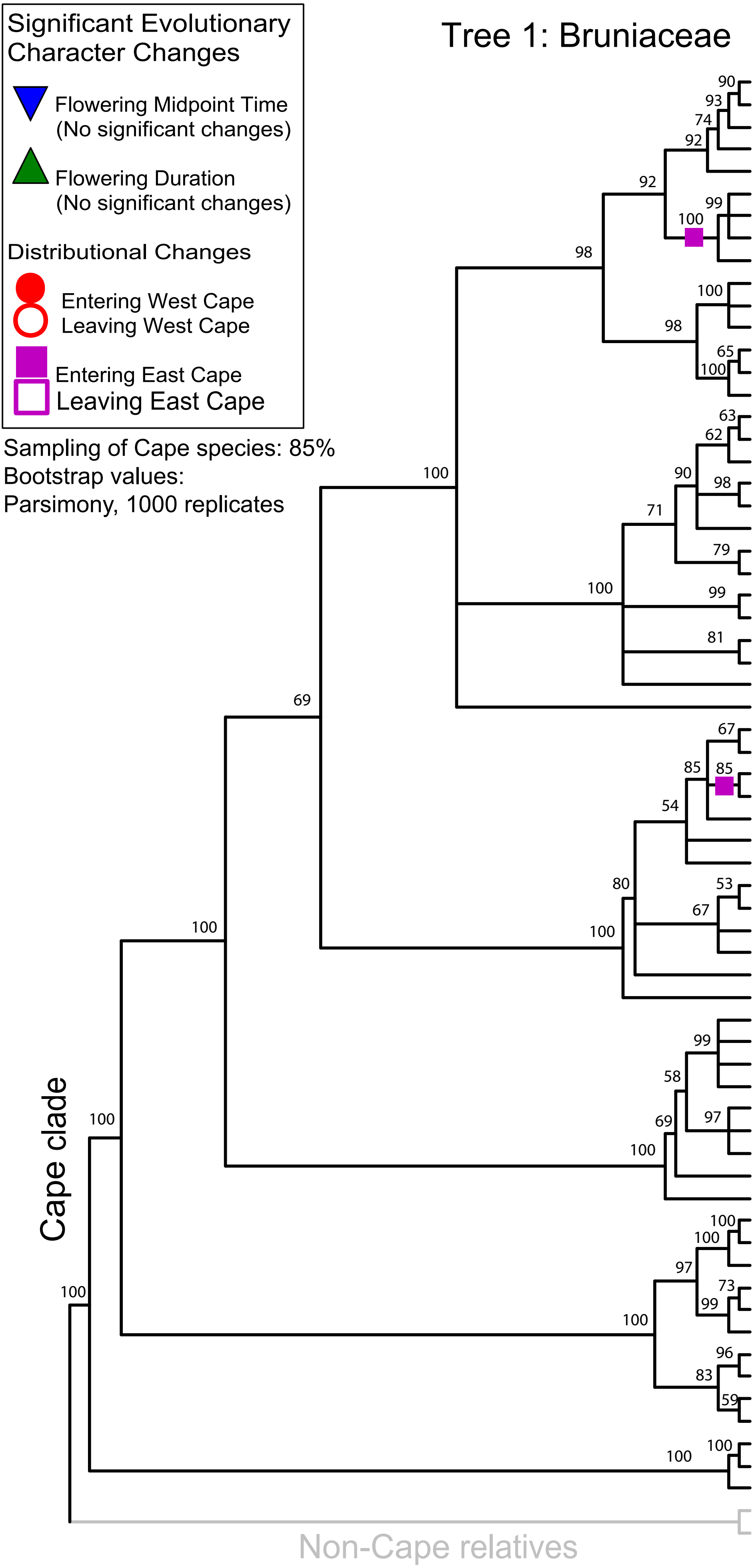
Significant Evolutionary Character Changes
Flowering Midpoint Time (No significant changes)
Flowering Duration (No significant changes)
Distributional Changes
Entering West Cape
Leaving West Cape
Entering East Cape
Leaving East Cape
Sampling of Cape species: 85%
Bootstrap values:
Parsimony, 1000 replicates
Tree 1: Bruniaceae
Cape clade
Non-Cape relatives

Tree 2: Cyperaceae

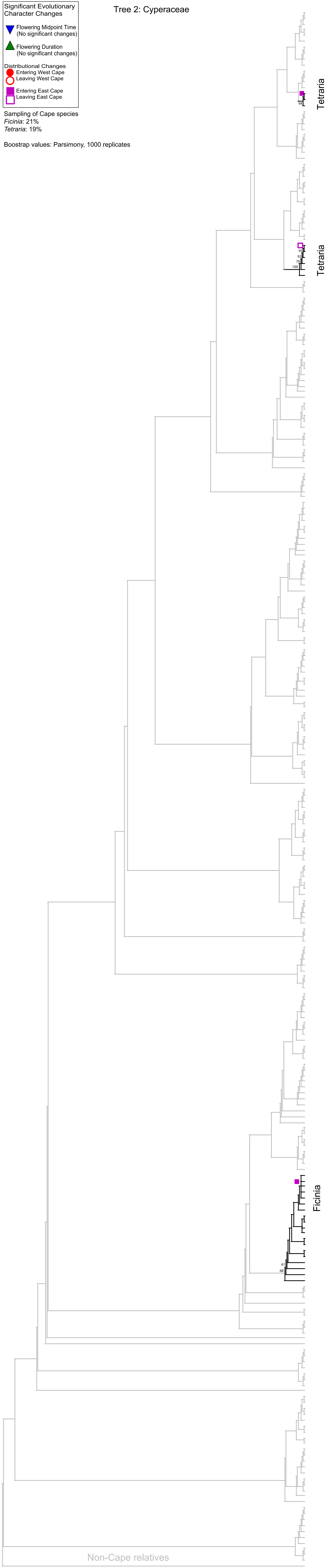

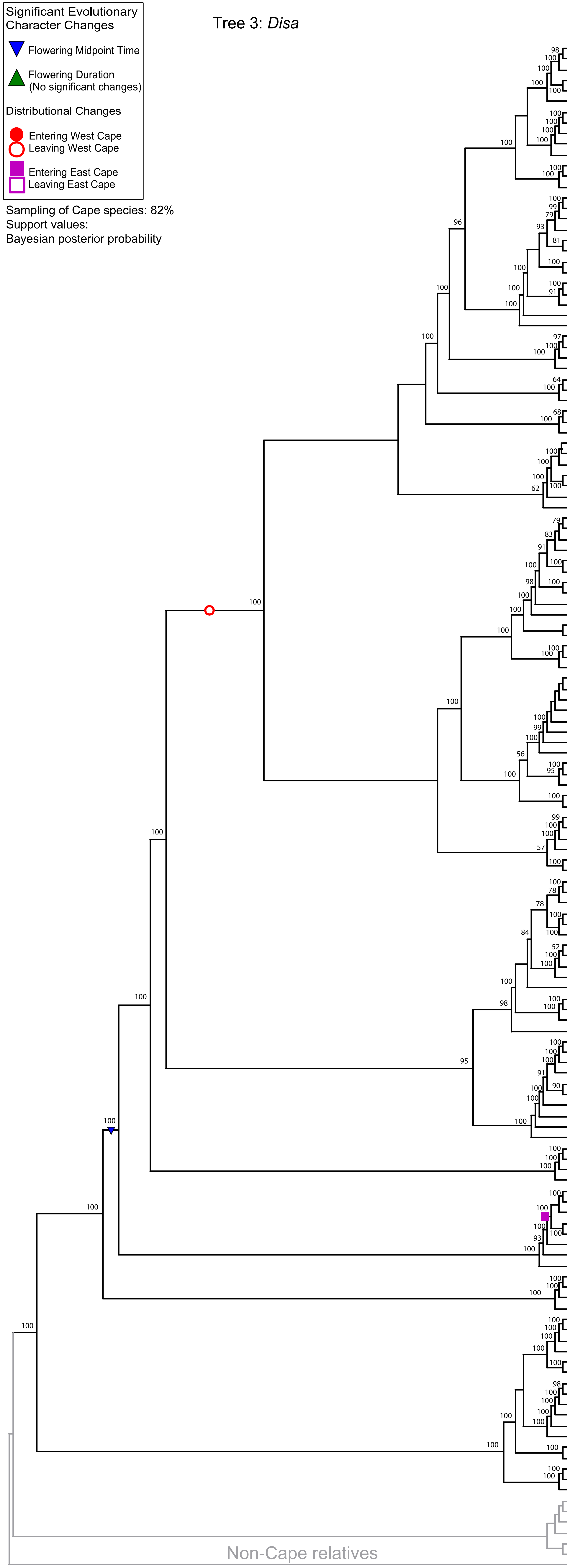

Tree 3: Disa
Significant Evolutionary Character Changes
Flowering Midpoint Time
Flowering Duration (No significant changes)
Distributional Changes
Entering West Cape
Leaving West Cape
Entering East Cape
Leaving East Cape
Sampling of Cape species: 82%
Support values:
Bayesian posterior probability
Non-Cape relatives

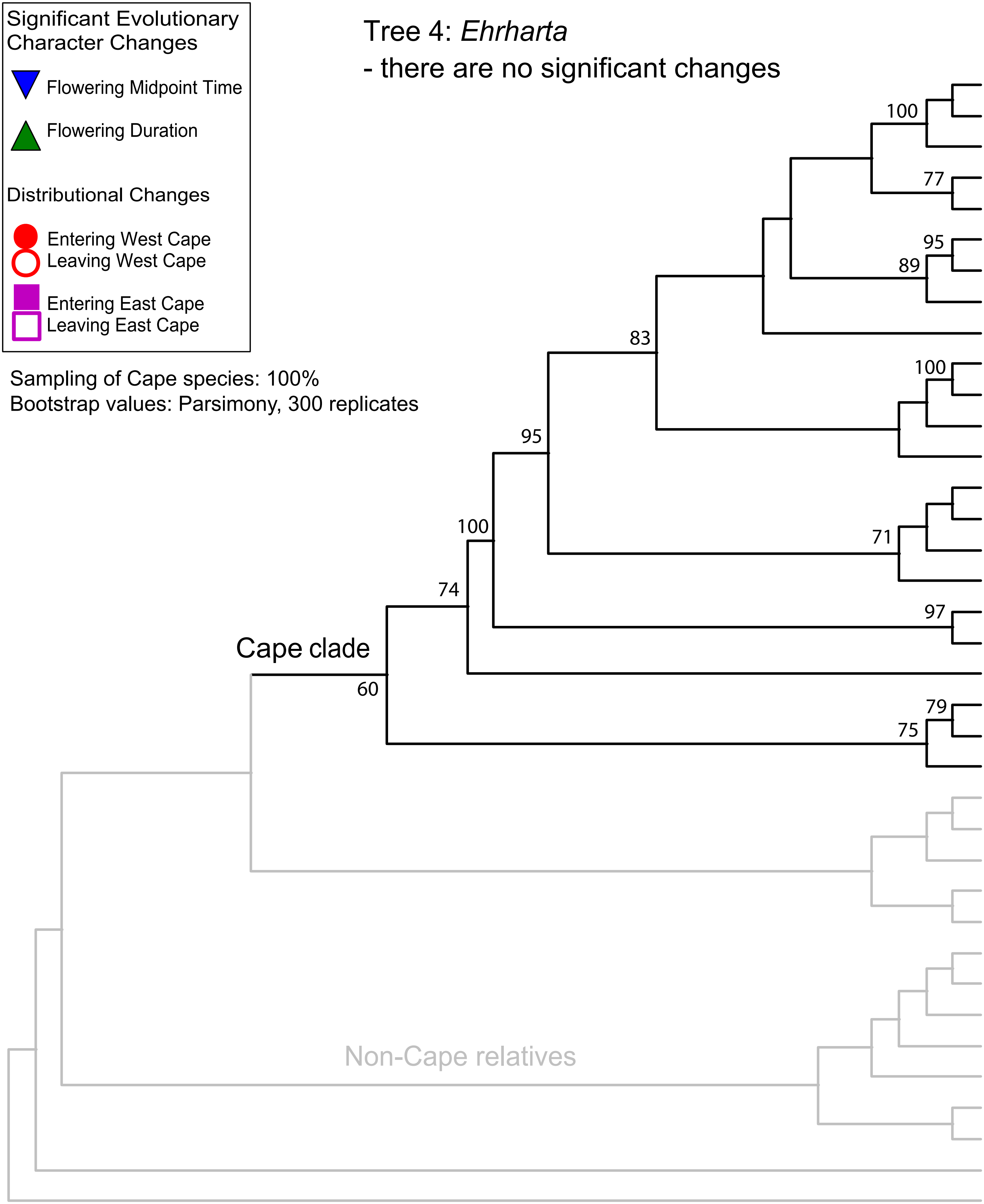
Significant Evolutionary Character Changes
Flowering Midpoint Time
Flowering Duration
Distributional Changes
Entering West Cape
Leaving West Cape
Entering East Cape
Leaving East Cape
Tree 4: Ehrharta
- there are no significant changes
Sampling of Cape species: 100%
Bootstrap values: Parsimony, 300 replicates
100
77
95
89
83
100
95
100
71
74
97
Cape clade
60
79
75
Non-Cape relatives

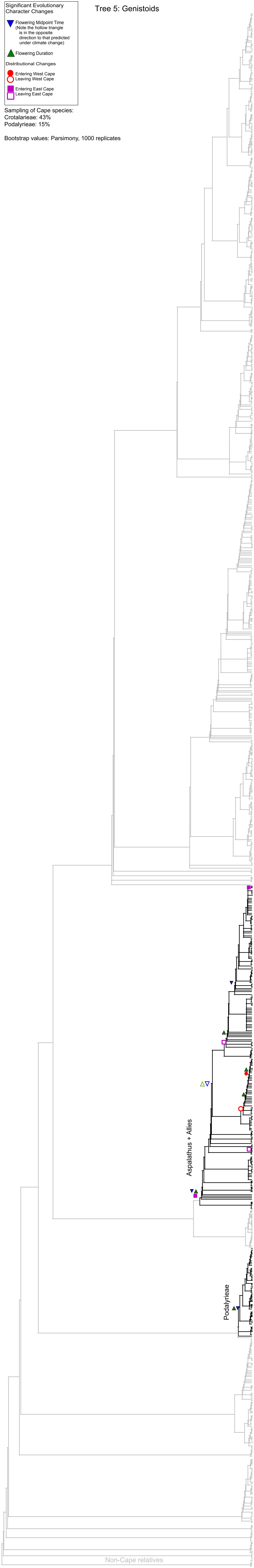
Tree 5: Genistoids
Significant Evolutionary Character Changes
Flowering Midpoint Time (Note the hollow triangle is in the opposite direction to that predicted under climate change)
Flowering Duration
Distributional Changes
Entering West Cape
Leaving West Cape
Entering East Cape
Leaving East Cape
Sampling of Cape species:
Crotalarieae: 43%
Podalyrieae: 15%
Bootstrap values: Parsimony, 1000 replicates
Aspalathus + Allies
Podalyrieae
Non-Cape relatives

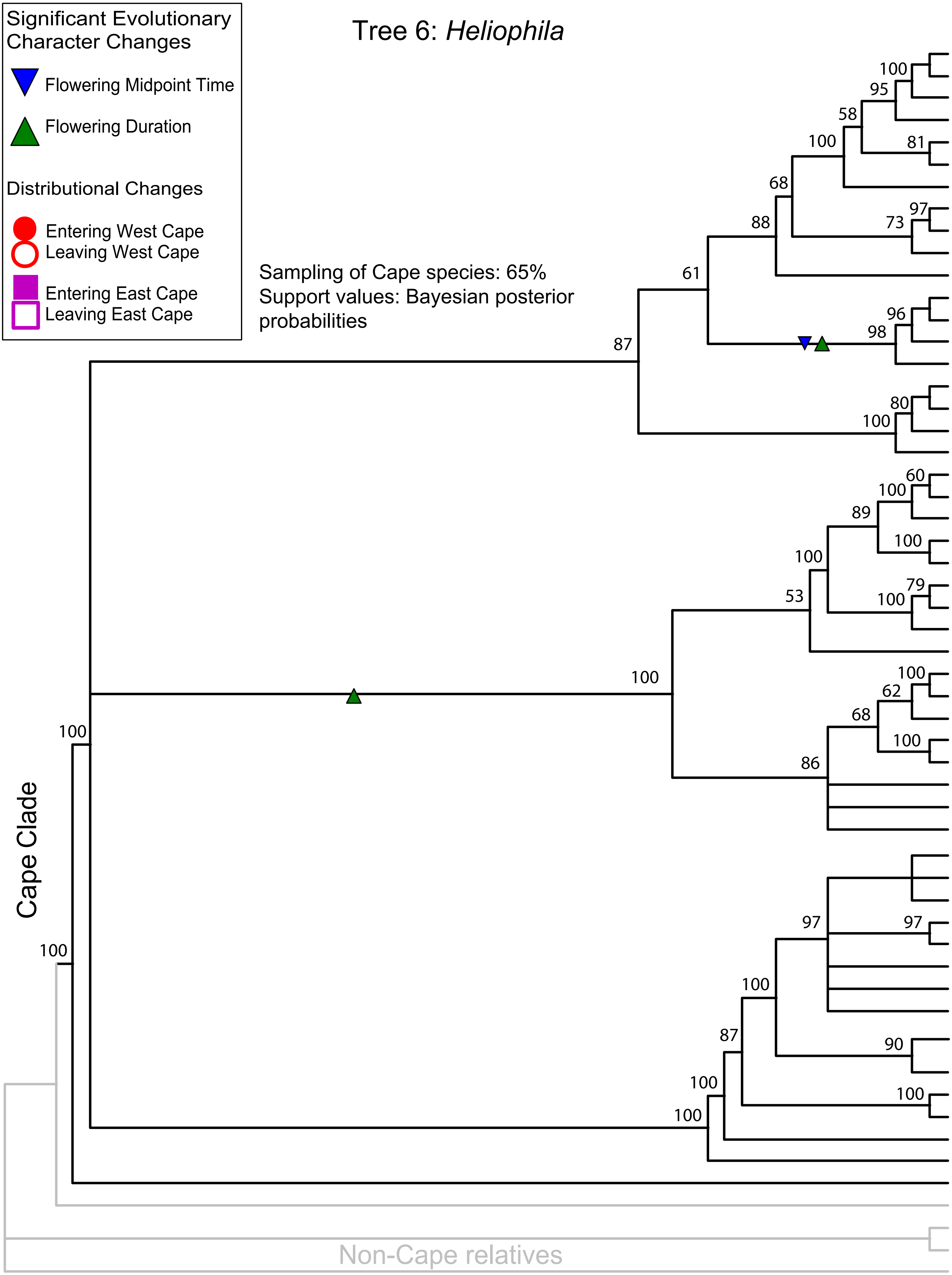
Tree 6: *Heliophila*
Significant Evolutionary Character Changes
Flowering Midpoint Time
Flowering Duration
Distributional Changes
Entering West Cape
Leaving West Cape
Entering East Cape
Leaving East Cape
Sampling of Cape species: 65%
Support values: Bayesian posterior probabilities
Cape Clade
Non-Cape relatives

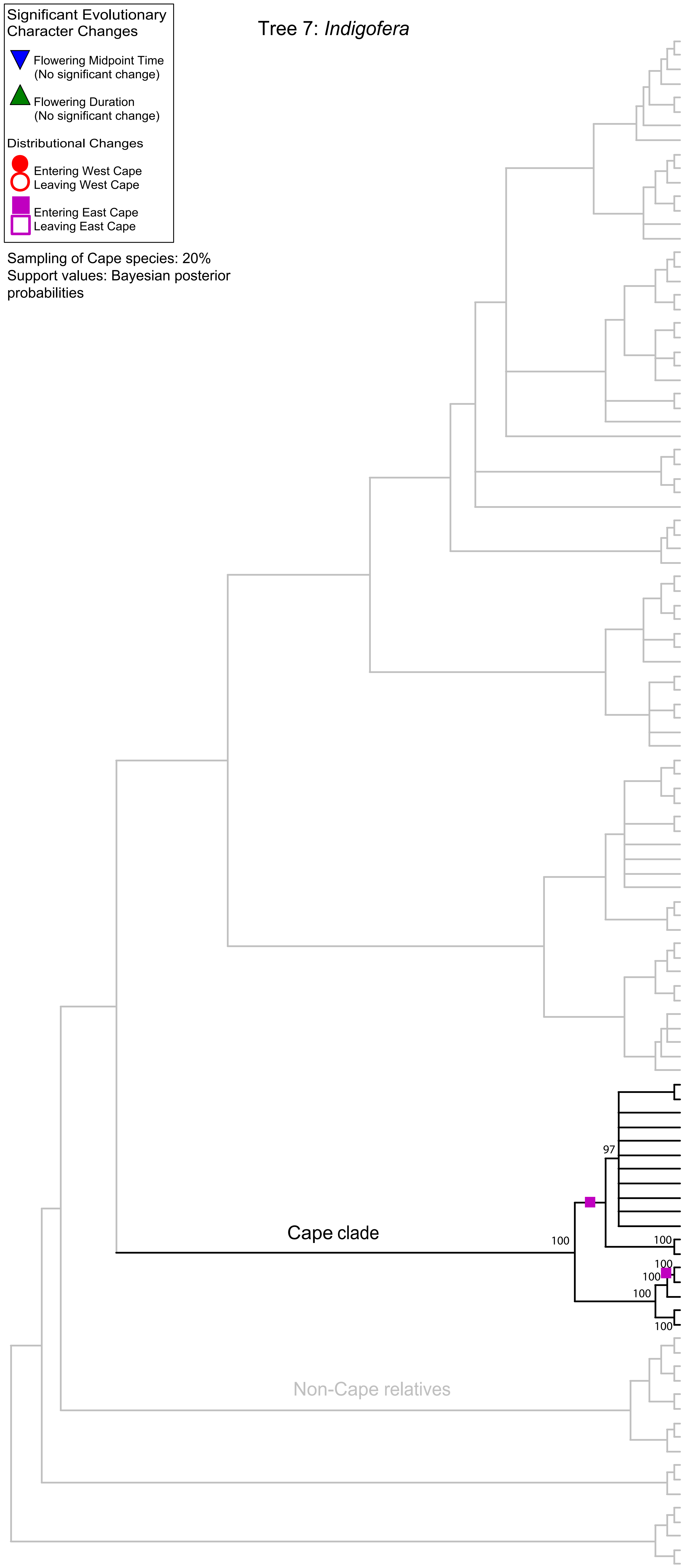

Tree 7: *Indigofera*
Significant Evolutionary Character Changes
Flowering Midpoint Time (No significant change)
Flowering Duration (No significant change)
Distributional Changes
Entering West Cape
Leaving West Cape
Entering East Cape
Leaving East Cape
Sampling of Cape species: 20%
Support values: Bayesian posterior probabilities
97
Cape clade
100
100
100
100
100
100
Non-Cape relatives

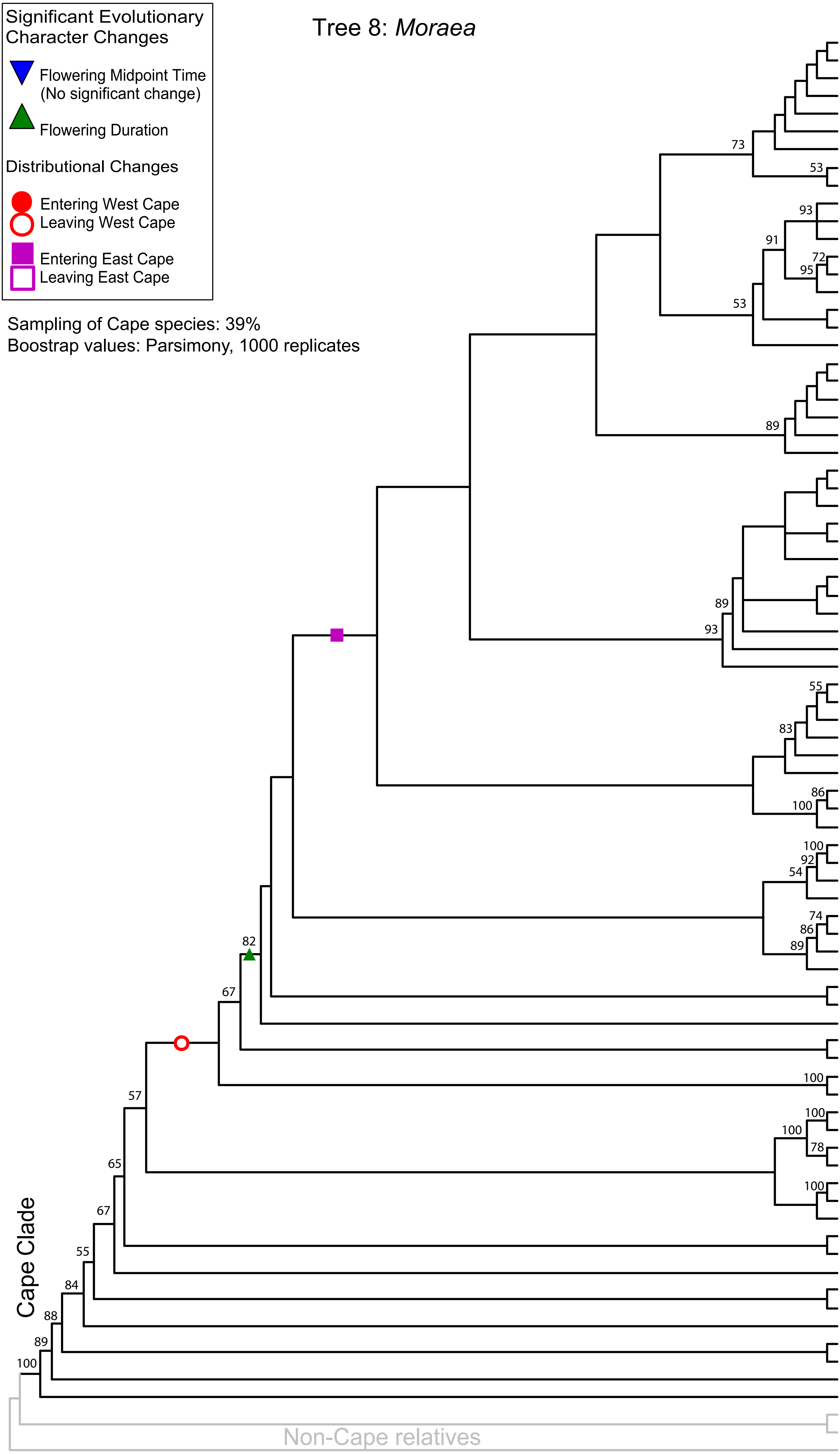

Tree 8: *Moraea*
Significant Evolutionary Character Changes
Flowering Midpoint Time (No significant change)
Flowering Duration
Distributional Changes
Entering West Cape
Leaving West Cape
Entering East Cape
Leaving East Cape
Sampling of Cape species: 39%
Boostrap values: Parsimony, 1000 replicates
Cape Clade
Non-Cape relatives

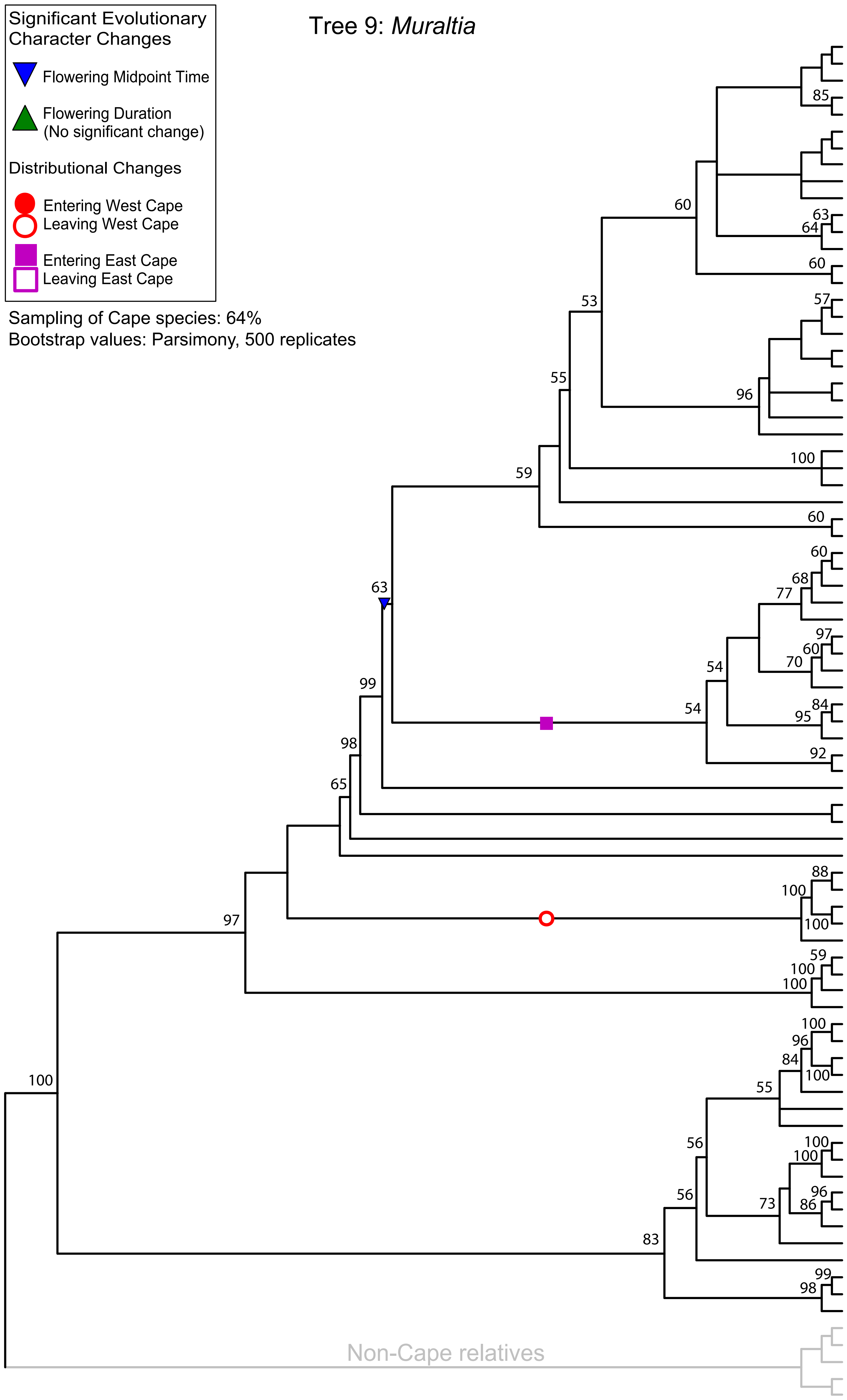
Tree 9: *Muraltia*
Significant Evolutionary Character Changes
Flowering Midpoint Time
Flowering Duration (No significant change)
Distributional Changes
Entering West Cape
Leaving West Cape
Entering East Cape
Leaving East Cape
Sampling of Cape species: 64%
Bootstrap values: Parsimony, 500 replicates
Non-Cape relatives

Tree 10: *Oxalis*

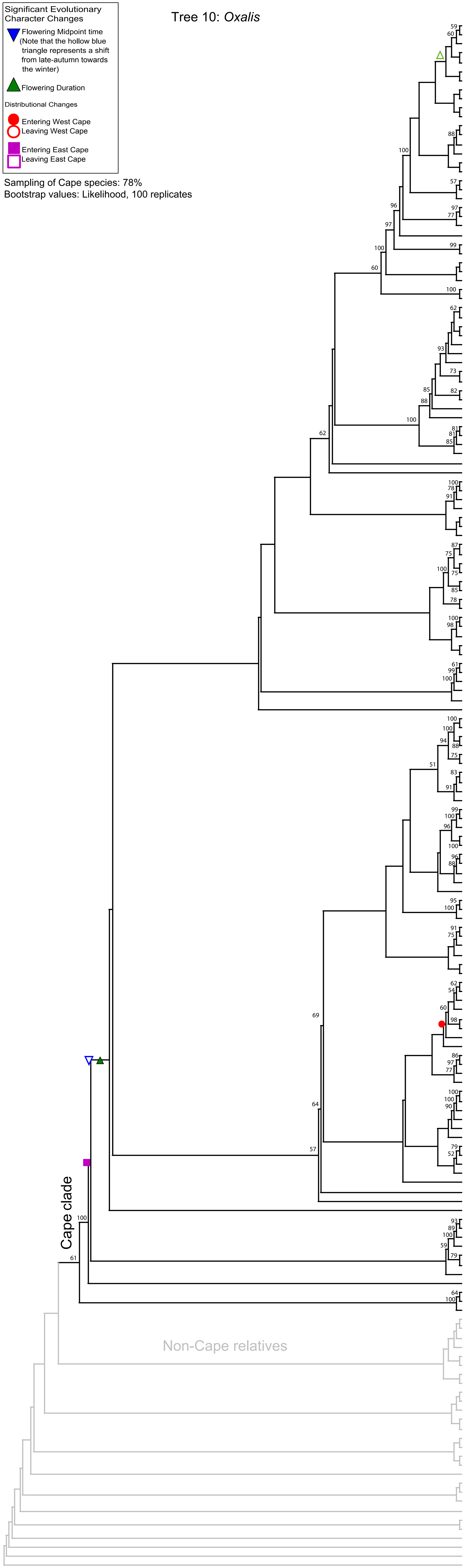

Tree 11: *Pelargonium*

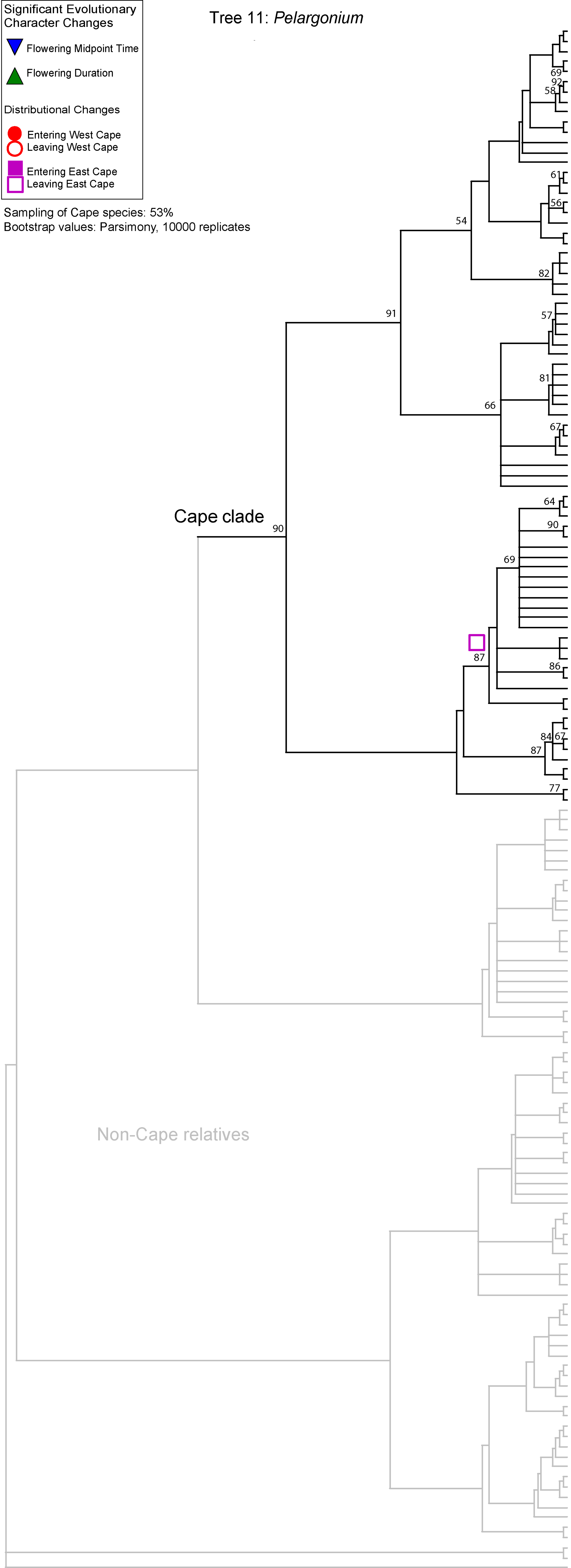

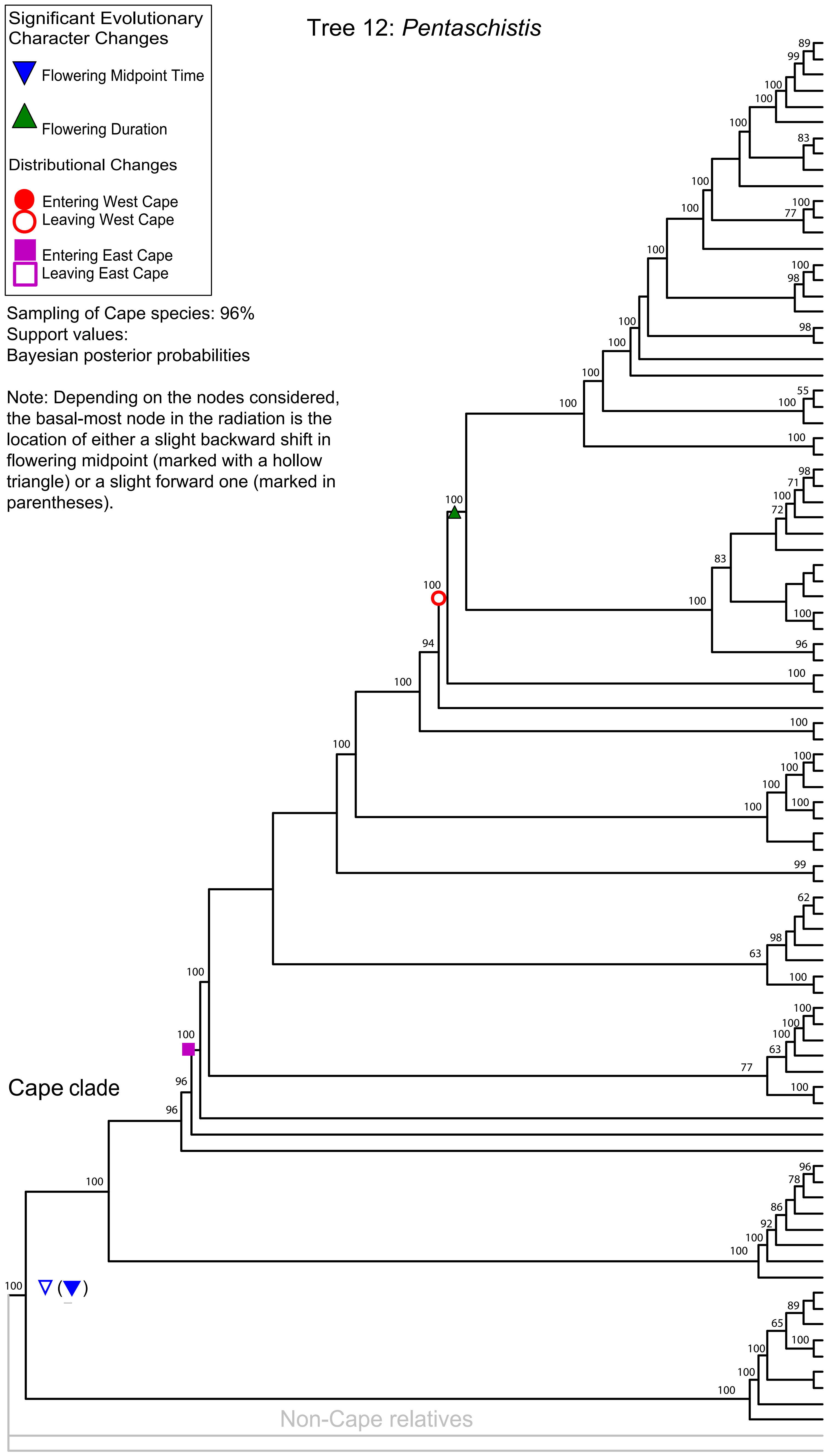
Tree 12: *Pentaschistis*
Significant Evolutionary Character Changes
Flowering Midpoint Time
Flowering Duration
Distributional Changes
Entering West Cape
Leaving West Cape
Entering East Cape
Leaving East Cape
Sampling of Cape species: 96%
Support values:
Bayesian posterior probabilities
Note: Depending on the nodes considered, the basal-most node in the radiation is the location of either a slight backward shift in flowering midpoint (marked with a hollow triangle) or a slight forward one (marked in parentheses).
Cape clade
Non-Cape relatives

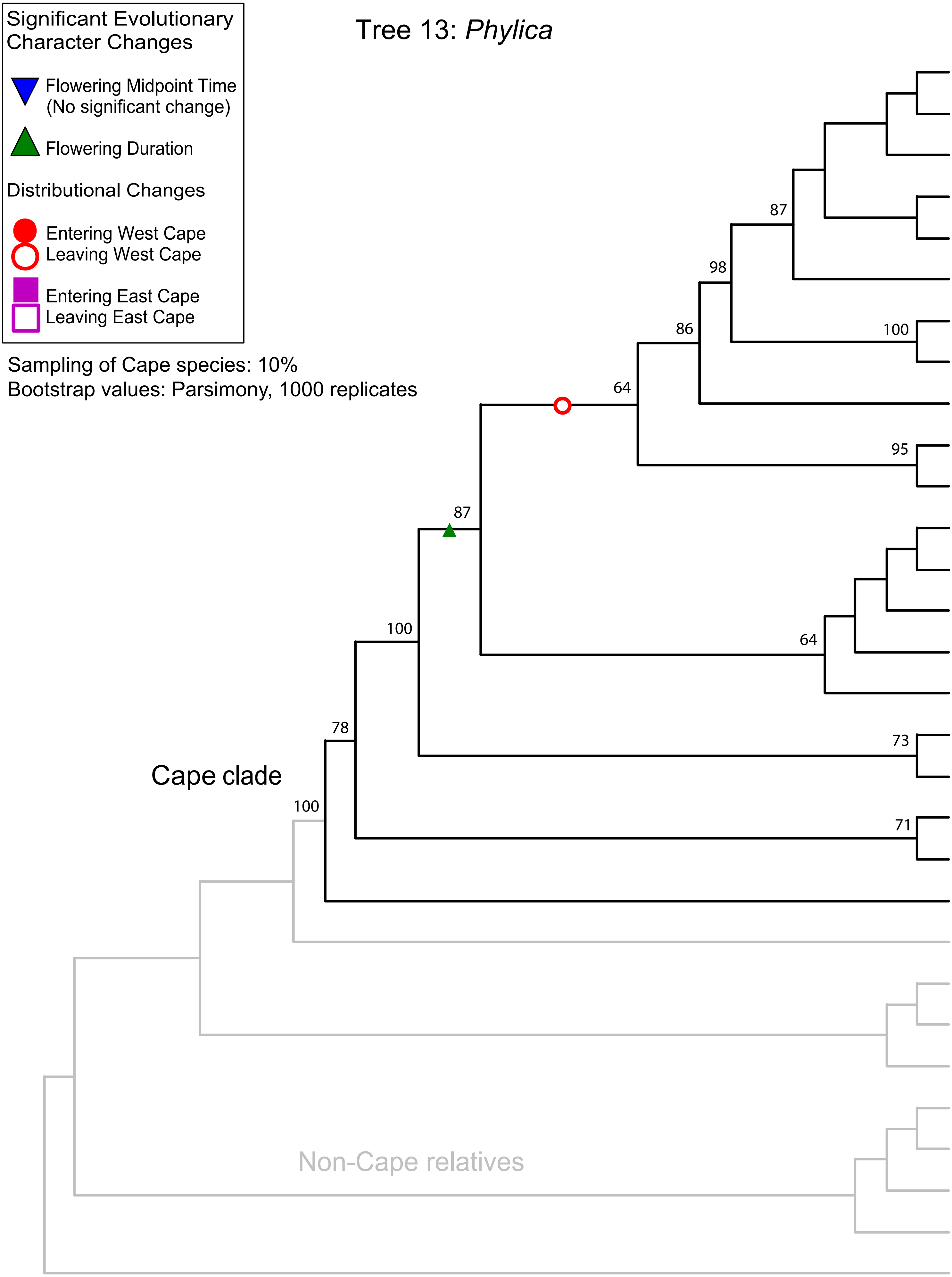

Significant Evolutionary Character Changes
Flowering Midpoint Time (No significant change)
Flowering Duration
Distributional Changes
Entering West Cape
Leaving West Cape
Entering East Cape
Leaving East Cape
Tree 13: *Phylica*
Sampling of Cape species: 10%
Bootstrap values: Parsimony, 1000 replicates
87
98
86
100
64
95
87
100
64
73
78
71
Cape clade
100
Non-Cape relatives

Significant Evolutionary Character Changes

- Flowering Midpoint Time (No significant changes)
- Flowering Duration

Distributional Changes

- Entering West Cape
- Leaving West Cape
- Entering East Cape
- Leaving East Cape

Sampling of Cape species: 89%
Bootstrap values: Parsimony

Note: Backward shifts in flowering are indicated with a hollow green triangle and precede forward shifts that concern much smaller numbers of species and are marked in parentheses.

Tree 14: Restionaceae

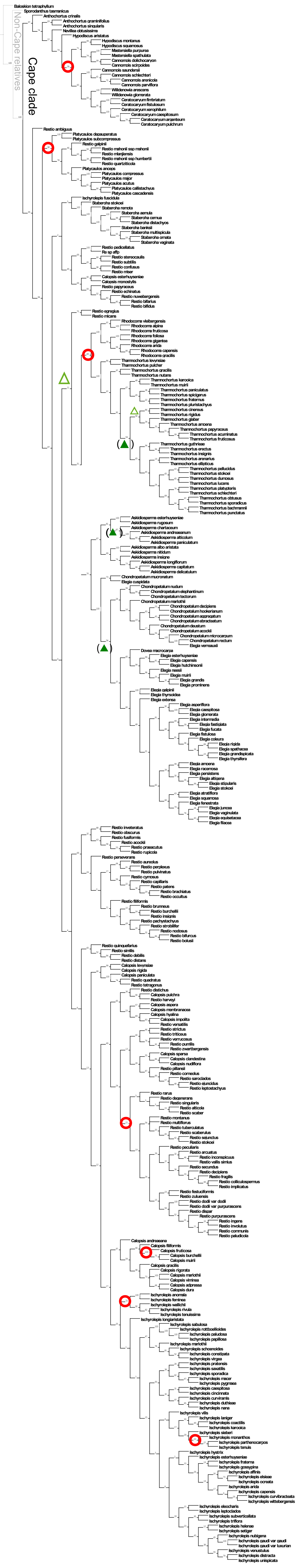

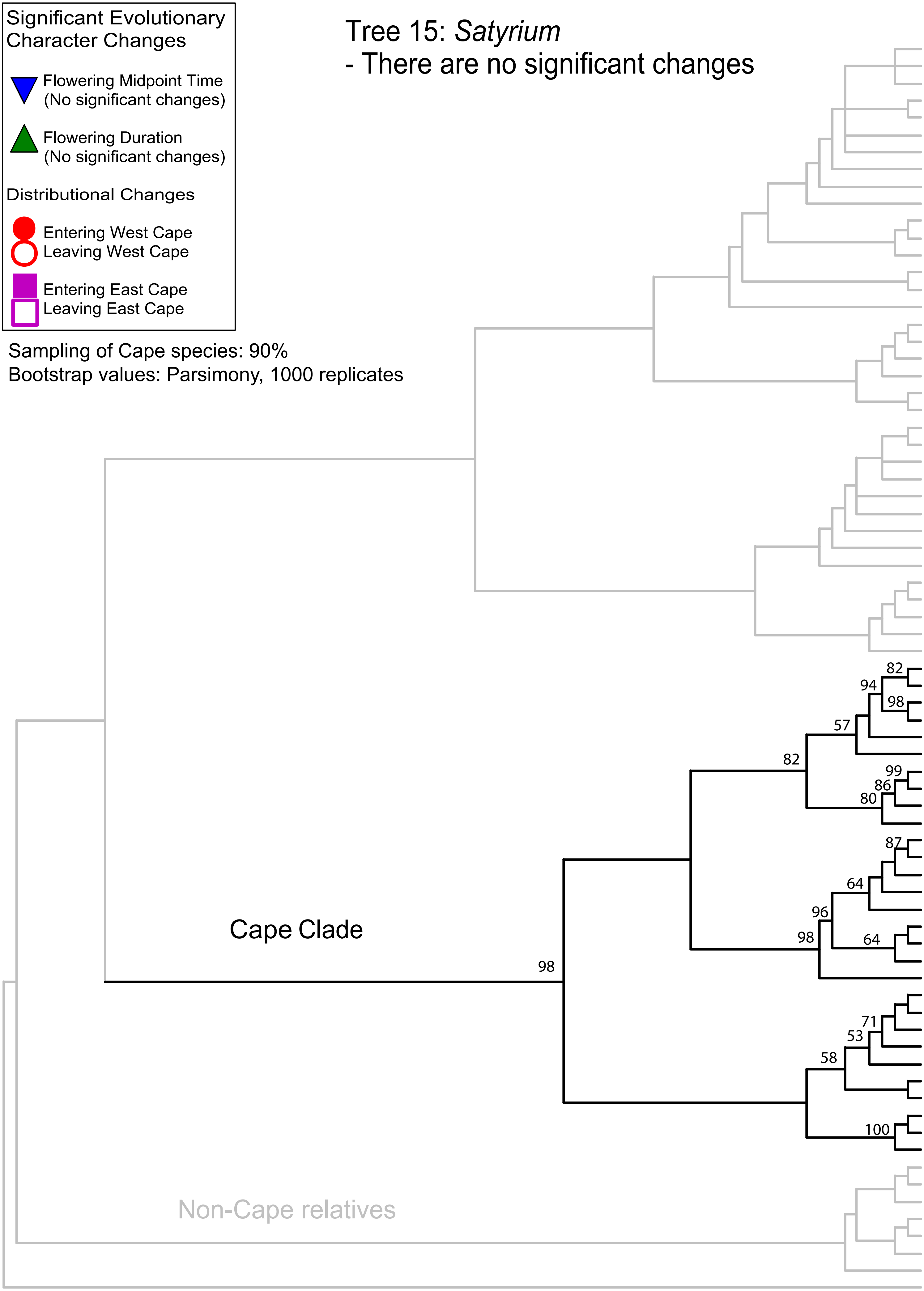
Tree 15: *Satyrium*
- There are no significant changes
Significant Evolutionary Character Changes
Flowering Midpoint Time (No significant changes)
Flowering Duration (No significant changes)
Distributional Changes
Entering West Cape
Leaving West Cape
Entering East Cape
Leaving East Cape
Sampling of Cape species: 90%
Bootstrap values: Parsimony, 1000 replicates
82
94
98
57
82
99
86
80
87
64
96
98
64
Cape Clade
98
71
53
58
100
Non-Cape relatives

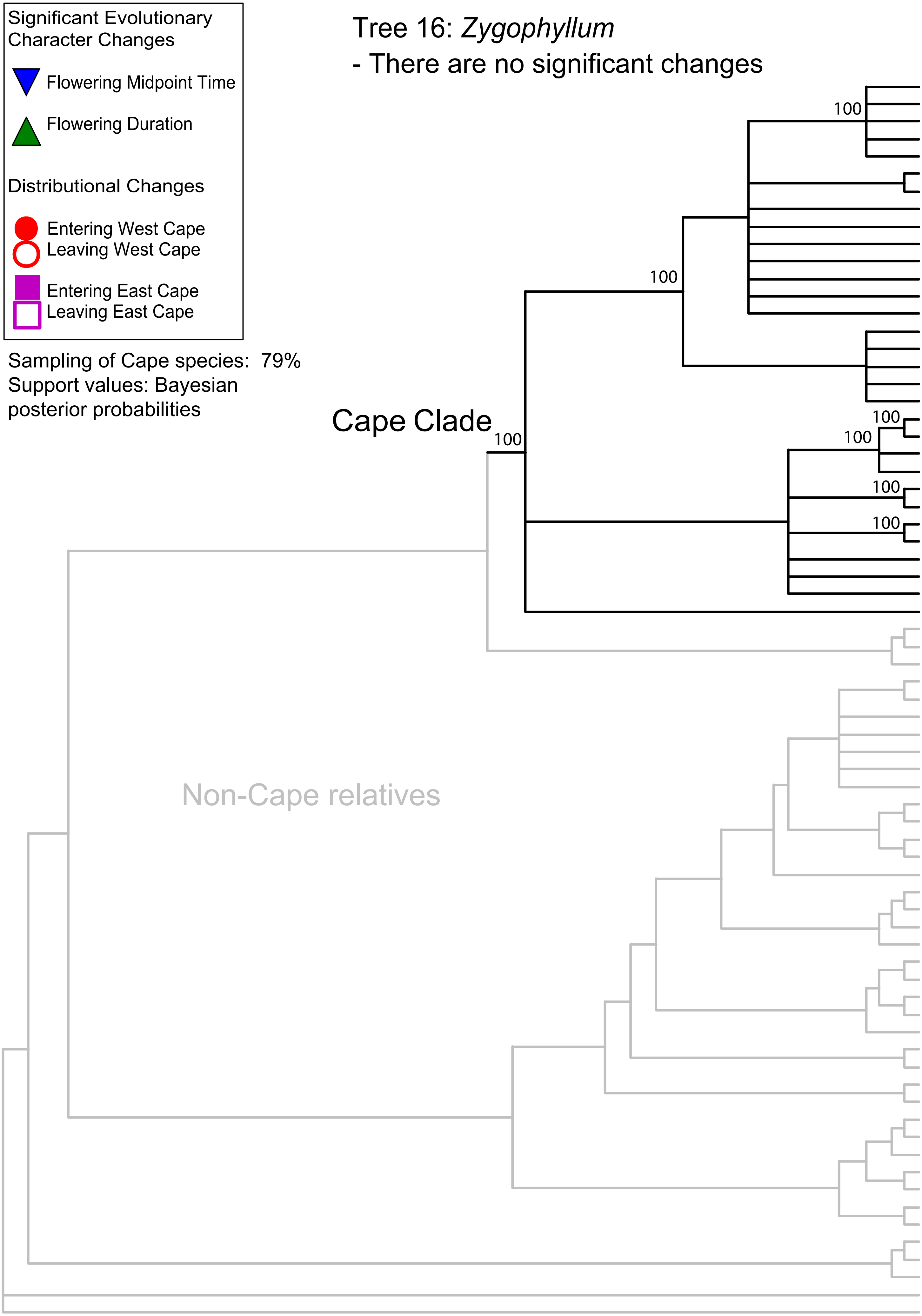

Significant Evolutionary Character Changes
Flowering Midpoint Time
Flowering Duration
Distributional Changes
Entering West Cape
Leaving West Cape
Entering East Cape
Leaving East Cape
Sampling of Cape species: 79%
Support values: Bayesian posterior probabilities
Tree 16: Zygophyllum
- There are no significant changes
100
100
Cape Clade
100
100
100
100
100
Non-Cape relatives
