# Supplementary material for: Consistent phenological shifts in the making of a biodiversity hotspot: the Cape flora
Source: BMC Evol Biol. 2011 Feb 8;11:39. doi: 10.1186/1471-2148-11-39 (PMC3045326; doi:10.1186/1471-2148-11-39)
Supplement: Additional file 2 — Table illustrating the degree of shift in flowering phenology in the eighteen Cape clades sampled. Mid-month flowering midpoint character states are indicated by an abbreviation for the month in question, while character states at the boundary between two months are indicated by those two month abbreviations separated by a hyphen. Flowering durations are in months. Shifts from the base of the tree towards the tips are indicated as ">". Note that all possible series in the degrees of shift of flowering midpoint and duration are listed. Many nodes optimised at different states are separated by nodes for which the ancestral state is undetermined. Therefore, how many shifts are counted depends on the criteria used to count them. In order to be conservative in our counting, we have counted shifts in the text, in Table 1 and in additional file AF1.pdf based on the basal-most possible location of each shift. As a result of these criteria and tree shape, there are many more possible series in the degrees of shifts marked here than there are basal-most possible positions of shifts in the text, Table 1 and file AF1.pdf. [file 1471-2148-11-39-S2.PDF]

Additional file 2 – Table illustrating the degree of shift in flowering phenology in the eighteen Cape clades sampled.

Mid-month flowering midpoint character states are indicated by an abbreviation for the month in question, while character states at the boundary between two months are indicated by those two month abbreviations separated by a hyphen. Flowering durations are in months. Shifts from the base of the tree towards the tips are indicated as “>”. Note that all possible series in the degrees of shift of flowering midpoint and duration are listed. Many nodes optimised at different states are separated by nodes for which the ancestral state is undetermined. Therefore, how many shifts are counted depends on the criteria used to count them.

In order to be conservative in our counting, we have counted shifts in the text, in Table 1 and in additional file AF1.pdf based on the basal-most possible location of each shift. As a result of these criteria and tree shape, there are many more possible series in the degrees of shifts marked here than there are basal-most possible positions of shifts in the text, Table 1 and file AF1.pdf.

Cape clade	Shift in flowering midpoint	Shift in flowering duration (months)
Bruniaceae		
Crotalarieae	Feb>Sept-Oct>Nov-Dec>Sept-Oct<br>Feb>Sept-Oct>Aug-Sept<br>Feb>Sept-Oct>Oct>Nov<br>Feb>Sept-Oct>Dec	9>4<br>9>6<br>9>4>5>4>8<br>9>4>12<br>9>4>3<br>9>4>2
Disa	Dec-Jan>Oct x2<br>Dec-Jan>Oct-Nov x3<br>Dec-Jan>Nov-Dec x3<br>Dec-Jan>Sept<br>Dec-Jan>Sept-Oct>Aug-Sept	
Ehrharta		
Ficinia		
Heliophila	Sept>Aug-Sept	3>2 x2
Indigofera		
Moraea		3>2 x3
Muraltia	Nov>Aug	
Oxalis	May>May-June x4<br>May>May-June>June<br>May>June	3>2 x5<br>3>4
Pelargonium		
Pentaschistis	Oct-Nov>Oct x3<br>Oct-Nov>Nov	2>1
Phylica		5>2
Podalyrieae	March>Jul-Aug	7>4
Cape Restionaceae		1>2>1 x2<br>1>2 x3<br>1>2>1>5
Satyrium		
Tetraria		
Zygophyllum		
